# Mean Kärger model water exchange rate in brain

**DOI:** 10.1162/imag_a_00335

**Published:** 2024-10-25

**Authors:** Jens H. Jensen, Joshua Voltin, Maria Fatima Falangola

**Affiliations:** Center for Biomedical Imaging, Medical University of South Carolina, Charleston, SC, United States; Department of Neuroscience, Medical University of South Carolina, Charleston, SC, United States; Department of Radiology and Radiological Science, Medical University of South Carolina, Charleston, SC, United States

**Keywords:** Kärger model, water exchange, diffusion, brain, kurtosis, mouse

## Abstract

Intercellular water exchange in brain is analyzed in terms of the multi-compartment Kärger model (KM), and the mean KM water exchange rate is used as a summary statistic for characterizing the exchange processes. Prior work is extended by deriving a stronger lower bound for mean exchange rate that can be determined from the time dependence of the diffusional kurtosis. In addition, an analytic formula giving the time dependence of the kurtosis for a model of thin cylindrical neurites is demonstrated, and this formula is applied to numerically test the accuracy of the lower bound for a range of model parameters. Finally, the lower bound is measured in vivo with diffusional kurtosis imaging for the dorsal hippocampus and cerebral cortex of 8-month-old mice. From the stronger lower bound, the mean KM exchange rate is found to be 46.1 ± 11.0 s^-1^or greater in dorsal hippocampus and 20.5 ± 8.5 s^-1^or greater in cortex.

## 1 Introduction

Modeling the effect of intercellular water exchange on the diffusion MRI (dMRI) signal in brain is challenging because of the brain’s microstructural complexity. In particular, the multiplicity of cell types with intricate morphologies, including relatively large soma and extensive thin neurites, produce a broad spectrum of exchange rates reflecting diverse exchange processes. Indeed, depending on the specific models and methods employed, experimental estimates for water exchange rates obtained from dMRI have produced widely varying results even in similar brain regions (Li et al., 2023).

Such experimental values can be viewed as mean or effective rates and may serve as useful summary statistics for characterizing key aspects water exchange in brain. However, established approaches are mainly based on simplified models (Jelescu et al., 2022;Lee et al., 2020;Meier et al., 2003;Mougel et al., 2024;Pfeuffer et al., 1998;Stanisz et al., 1997;Uhl et al., 2024;Zhang et al., 2021), typically having only two or three compartments, or are essentially empirical without a clear underlying physical picture (Bai et al., 2020;Lampinen et al., 2017;Nilsson et al., 2013;Shin et al., 2024). As a consequence, the precise connections of measured rates to the full exchange dynamics of brain microstructure are often obscure. The discrepant results obtained in prior work could be due, at least in part, to differences in these connections. Moreover, application of highly simplified models or empirical techniques can make measurements more prone to systematic bias and thereby also increase variability.

Recently, a mean exchange rate,$R_{\text{KM}}$, has been introduced for the Kärger model (KM;Kärger et al., 1988) of water exchange with any number of compartments, arbitrary compartmental diffusivities, and arbitrary intercompartmental exchange rates (Jensen, 2024). In addition, a practical experimental method has been proposed for obtaining a lower bound on$R_{\text{KM}}$from the time dependence of the diffusional kurtosis.$R_{KM}$has a well-defined physical interpretation for any KM, and it may thus support a more reproducible and robust assessment of the brain’s water exchange properties.

To be sure, the KM is only an approximate description of water exchange as it neglects intracellular structure by treating diffusion as random hopping of water molecules between compartments in which the diffusion is otherwise unrestricted. However, it is expected to be valid under conditions that are plausible for some dMRI experiments performed in brain. Specifically, the diffusion time and the intracellular residence times should all be long or comparable to the time required for a water molecule to explore the diffusion landscape within each compartment, which would typically be about$L_{n}^{2}/D_{n}$for a compartment with a length scale$L_{n}$and an intracompartmental diffusivity$D_{n}$(Fieremans et al., 2010). So, for a 4 µm compartment with a diffusivity of 2 µm^2^/ms, the diffusion and residence times should be at least 8 ms. A strong, if not definitive, experimental test of the applicability of the KM is given by the time dependence of the total diffusivity, which is predicted to be constant for any KM (Jensen, 2024). Nearly constant diffusivities have been observed in the cortex for diffusion times exceeding about 10 ms, supporting the validity of the KM in this region (Aggarwal et al., 2020;Jelescu et al., 2022;Lee et al., 2020;Pyatigorskaya et al., 2014;Uhl et al., 2024).

In this paper, we extend prior work on$R_{\text{KM}}$in three ways. First, we derive an enhancement factor,$E_{f}$, that strengthens the previously derived lower bound. Second, we give an analytic formula for the time dependence of the kurtosis in a system comprising thin cylindrical cells, which have often been used for modeling water diffusion within neurites, and apply this formula to numerically investigate how accurately$R_{\text{KM}}$is predicted by the lower bound. Third, we present new data acquired in vivo at 7 T for the time dependence of the kurtosis in the dorsal hippocampus (DH) and cerebral cortex (CX) of 8-month-old mice, which are used to derive lower bounds on$R_{\text{KM}}$for these two brain regions.

## 2 Theory

### 2.1 Definition of mean KM water exchange rate

To analyze the multi-compartment KM, it is convenient to express the defining equations in matrix form, as discussed in detail in prior work (Jensen, 2024). Briefly, the multi-compartment KM can be written as

$$\frac{\partial }{\partial t}F\left(x,t\right)=D\frac{\partial^{2}}{\partial x^{2}}F\left(x,t\right)+GF\left(x,t\right)$$ (1)

with the initial condition

$$F\left(x,0\right)=\sigma^{-1\;}\delta \left(x\right).$$ (2)

InEquations 1and2,Dis the diffusivity matrix,σis the water fraction matrix,δ(x)is the Dirac delta function,$F(x,t)=\sigma^{-1\;}P(x,t)$, whereP(x,t)is the diffusion displacement probability density matrix, and$G=\sigma^{-1\;}R\sigma$, whereRis the exchange rate matrix. The matricesD**,**F,σ,P,G, andRare allN×N, whereN>1is the number of compartments. The components ofP(x,t)are$P_{mn}(x,t)$and give the diffusion displacement probability density for a water molecule moving a distancexover a time intervaltwhile hopping from compartmentnto compartmentm. The components ofσare$\sigma_{mn}=\sqrt{f_{m}}\delta_{mn}$, where$f_{m}$is the water fraction for themth compartment, and$\delta_{mn}$is the Kronecker delta. The components$R_{mn}$of the exchange rate matrix give the transition rate from compartmentnto compartmentm, and the components of the diffusivity matrix are$D_{mn}=D_{m}\delta_{mn}$, where$D_{m}$is the diffusivity inside compartmentm. Typically,$D_{m}$would be an effective diffusivity that could depend on the microstructural details in perhaps an unspecified way. The water fractions are normalized so that$\sum_{m=1}^{N}f_{m}=1$. The matrixGmust always be symmetric and negative semi-definite. Therefore, it hasNorthonormal eigenvectors,$\nu_{n}$, withNassociated eigenvalues$\lambda_{n}$, which all must be zero or negative. We order the eigenvalues so that$\lambda_{n}\geq \lambda_{n+1}$. In all cases,$\lambda_{1}=0$with the corresponding eigenvector$\nu_{1}=\eta$, whereηhas the components$\eta_{m}=\sqrt{f_{m}}$. The appearance of$\sqrt{f_{m}}$is a natural consequence of bringing the KM equations into a symmetrized form (Jensen, 2024;Jensen & Helpern, 2011). Forn>1, the eigenvectors$\nu_{n}$usually depend on both the water fractions and the components of the exchange rate matrix, and they reflect combinations of water fraction perturbations that decay monoexponentially to equilibrium rather than individual physical compartments.

The mean KM water exchange rate is then defined by

$$R_{\text{KM}}\equiv \sum_{n=2}^{N}\frac{\kappa_{n}}{K_{0}\tau_{n}},$$ (3)

where$K_{0}$is the total diffusional kurtosis in the limit of zero diffusion time,$\kappa_{n}$is the partial initial kurtosis associated with$\nu_{n}$, and$\tau_{n}=-1/\lambda_{n}$is an exchange time associated with$\nu_{n}$. The partial kurtosis is given explicitly by

$$\kappa_{n}=\frac{3}{D^{2}}{\left|\eta^{T}D\nu_{n}\right|}^{2}\geq 0,$$ (4)

where

$$D\equiv \sum_{n=1}^{N}f_{n}D_{n}\;$$ (5)

is the total diffusivity of the system. InEquation 3, terms of the sum for which$\lambda_{n}=0$automatically vanish since$\tau_{n}$is then infinite.

One can show that

$$K_{0}=\sum_{n=2}^{N}\kappa_{n}=\frac{3\delta^{2}D}{D^{2}},$$ (6)

where$\delta^{2}D$is the variance of the compartmental diffusivities. Therefore, the factor$\kappa_{n}/K_{0}$appearing inEquation 3can be interpreted as the fractional contribution of$\nu_{n}$to the total initial kurtosis. The mean KM water exchange rate is then a kurtosis-weighted average of the inverse exchange times, which gives it a well-defined meaning for any KM.

FromEquation 6, it is evident that$K_{0}$is simply 3 times the square of the coefficient of variation for the distribution of compartmental diffusivities and is therefore a measure of the system’s diffusional heterogeneity. The contribution of each eigenvector to this heterogeneity is thus given precisely by the partial kurtosis. Since the terms on the right side ofEquation 3are weighted by this contribution,$R_{\text{KM}}$reflects exchange processes between dissimilar diffusion compartments more strongly than between similar compartments.

### 2.2 Diffusion elasticity

A major prediction of the KM is that the total diffusivityDis independent of time as indicated byEquation 5. Thus, the diffusivity’s time dependence provides a convenient means of testing the validity of the KM for any particular dataset. A dimensionless measure for the strength of the time dependence is given by the diffusion elasticity defined as

$$\xi \left(t\right)\equiv t\frac{d}{dt}\left\{\ln\left[D\left(t\right)\right]\right\}.$$ (7)

The concept of elasticity is used in chemical kinetics (Woods & Sauro, 1997) and economics (Nievergelt, 1983) to quantify the sensitivity of one parameter on another. The diffusion elasticity should never be positive since the diffusivity cannot increase with time for a system in equilibrium. If its magnitude is much less than one, then it is reasonable to regard the diffusivity as being nearly constant as predicted by the KM. In practice, the diffusion elasticity can be estimated directly from the slope of the diffusivity as a function of time on a log-log plot. For$D\left(t\right)\propto t^{-\alpha }$, one simply hasξ=−α.

### 2.3 Lower bound

We have previously shown that

$$R_{\text{KM}}\geq \max^{\text{*}}\left\{-3\frac{d}{dt}\ln\left[K\left(t\right)\right]\right\}\equiv R_{\text{KM}}^{*},$$ (8)

whereK(t)is the diffusional kurtosis as a function of time and the maximum is taken over all available times judged as consistent with the KM (Jensen, 2024). The asterisk superscript is appended to the max function to indicate this restriction to times for which the KM is considered valid. Since the KM kurtosis can be shown to be logarithmically convex and monotonically decreasing, the optimal times for evaluating the logarithmic derivative appearing inEquation 8are the shortest of the KM consistent diffusion times.

The time selected to determine$R_{\text{KM}}^{*}$will be called$t^{*}$. For an experiment, this would actually refer to a small range of diffusion times, centered around$t^{*}$, sufficient for estimating the slope ofln[K(t)]. In many cases, one would use the data with the shortest available diffusion times in order to obtain the tightest bound. However, one may sometimes wish to exclude very short diffusion times if these are not long enough for KM behavior to fully manifest (Fieremans et al., 2010;Jensen, 2024).

### 2.4 Enhancement factor

Based on numerical simulations discussed in our prior work (Jensen, 2024), we speculated on the possibility of a stronger lower bound for$R_{KM}$than provided byEquation 8. That conjecture can indeed be proven correct and expressed as

$$R_{\text{KM}}\geq {\hat{R}}_{\text{KM}}\equiv E_{f}\cdot R_{\text{KM}}^{*}\geq R_{\text{KM}}^{*},$$ (9)

where$E_{f}\geq 1$is an enhancement factor that generates an improved bound${\hat{R}}_{\text{KM}}$. The enhancement factor is given explicitly by

$$E_{f}=V\left(R_{\text{KM}}^{*}t^{*}\right)$$ (10)

and

$$V(x)=\frac{1}{x}\beta^{-1}(x)$$ (11)

with

$$\beta \left(x\right)\equiv 3\frac{x-2+\left(x+2\right)e^{-x}}{x-1+e^{-x}}.$$ (12)

By applying the Lagrange inversion theorem (Abramowitz & Stegun, 1972), we find the expansion

$$\begin{array}{l} V\left(x\right)=1+\frac{1}{6}x+\frac{2}{45}x^{2}+\frac{7}{540}x^{3}+\frac{113}{28350}x^{4}\\ +\frac{43}{34020}x^{5}+\frac{149}{364500}x^{6}+O\left(x^{7}\right).\; \end{array}$$ (13)

A plot of the functionV(x)is given byFigure 1Atogether with the approximation ofEquation 13, which is accurate to within 3% forx<2. Forx>3,V(X)is undefined, but this is physically irrelevant since$R_{\text{KM}}^{*}t^{*}\leq 3$for any KM (Jensen, 2024). At the pointx=3,V(x)is singular, in which caseEquation 10does not apply. A proof ofEquation 9is sketched in theAppendix.

**Fig. 1.**
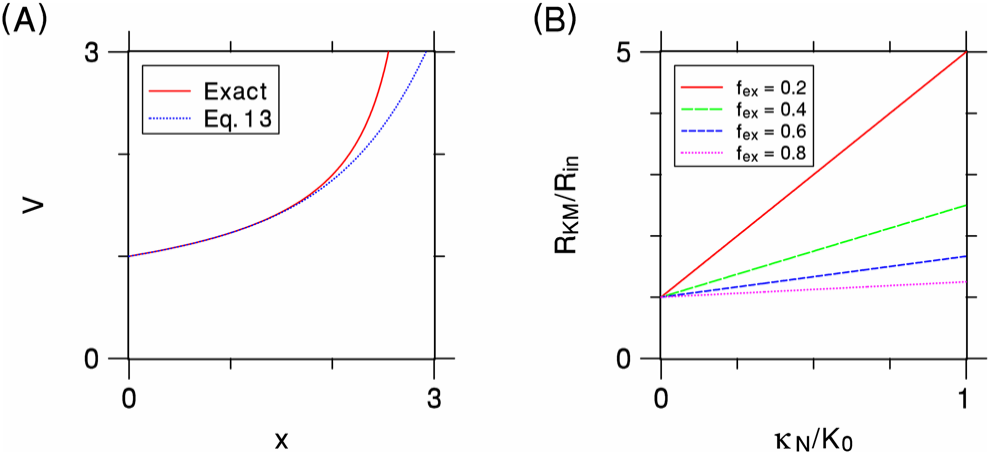
(A) The functionV(x)needed to calculate the enhancement factor$E_{f}$. The solid line shows the exact form given byEquations 11and12while the dotted line is the sixth order approximation ofEquation 13.V(x)is only defined for0≤x<3, which is the range of physical interest. (B) Ratio$R_{KM}/R_{\text{in}}$as a function of$\kappa_{N}/K_{0}$for several values of the extra-neurite water fraction$f_{ex}$in the thin cylindrical neurite model. If$\kappa_{N}/K_{0}\ll f_{\text{ex}}/(1-f_{\text{ex}})$, then$R_{KM}\approx R_{\text{in}}$, but$R_{KM}$may be substantially larger than$R_{\text{in}}$otherwise. However,$R_{\text{in}}\leq R_{KM}\leq R_{\text{in}}/f_{\text{ex}}$in all cases.

For the special caseN=2, we always have$R_{\text{KM}}={\hat{R}}_{\text{KM}}$. Indeed, one can interpret${\hat{R}}_{\text{KM}}$as$R_{\text{KM}}$for a two-compartment KM model in which the predictedln[K(t)]curve is tangent to the experimentalln[K(t) ​]at$t=t^{*}$. The argument in theAppendixshows that the two-compartment$R_{\text{KM}}$estimated in this way will always be a lower bound for the true$R_{\text{KM}}$regardless of the actual number of compartments. Thus, one obtains a prediction that applies to all KMs.

### 2.5 Thin cylindrical neurite model

The kurtosis for any KM is given byJensen (2024)

$$K\left(t\right)=\sum_{n=2}^{N}\kappa_{n}ϒ\left(\frac{t}{\tau_{n}}\right),$$ (14)

where

$$ϒ\left(x\right)\equiv 2\overset{1}{\underset{0}{\int }}ds\left(1-s\right)e^{-sx}=\frac{2}{x}\left(1-\frac{1-e^{-x}}{x}\right).$$ (15)

As a specific example, we now consider a system consisting of an ensemble of$N_{c}$identical thin cylindrical neurites. The*m*th neurite is assumed to be oriented at an angle$\theta_{m}$relative to the diffusion direction of interest, which in practice is given by the direction of the diffusion gradient. The intracompartmental diffusivity for the*m*th neurite is then$D_{\text{in}}\cos^{2}\theta_{m}$, where$D_{\text{in}}$is the intrinsic intracompartmental diffusivity taken to be the same for all neurites. Direct water exchange is only allowed between the neurites and the extra-neurite space, which is assumed to have a diffusivity$D_{\text{ex}}$. The total number of compartments is then$N=\;N_{c}+1$. We designate the extra-neurite space as compartment 1 so that$D_{1}=D_{\text{ex}}$and$f_{1}=f_{\text{ex}}$, where$f_{\text{ex}}$is the water fraction for the extra-neurite space. Furthermore,$D_{m+1\;}=D_{\text{in}}\cos^{2}\theta_{m}$for$m=1,\ldots ,N_{c}$, and$f_{\text{in}}=\sum_{m=1}^{N_{c}}f_{m+1}$, where$f_{\text{in}}=1-f_{\text{ex}}$is the water fraction for the full set of neurites.

Water exchange processes for this model are governed by theN×Nsymmetric matrix

$$\text{G}=\left(\begin{array}{cccccc} -\frac{f_{in}}{f_{ex}}R_{in} & \sqrt{\frac{f_{in}}{N_{c}f_{ex}}}R_{in} & \sqrt{\frac{f_{in}}{N_{c}f_{ex}}}R_{in} & \sqrt{\frac{f_{in}}{N_{c}f_{ex}}}R_{in} & \cdots & \sqrt{\frac{f_{in}}{N_{c}f_{ex}}}R_{in}\\ \sqrt{\frac{f_{in}}{N_{c}f_{ex}}}R_{in} & -R_{in} & 0 & 0 & \cdots & 0\\ \sqrt{\frac{f_{in}}{N_{c}f_{ex}}}R_{in} & 0 & -R_{in} & 0 & \cdots & 0\\ \sqrt{\frac{f_{in}}{N_{c}f_{ex}}}R_{in} & 0 & 0 & -R_{in} & \vdots & \vdots \\ \vdots & \vdots & \vdots & \cdots & \ddots & 0\\ \sqrt{\frac{f_{in}}{N_{c}f_{ex}}}R_{in} & 0 & 0 & \cdots & 0 & -R_{in} \end{array}\right)$$ (16)

where$R_{\text{in}}$is the rate for water molecules leaving an individual neurite. The first eigenvector forGis

$$\nu_{1}=\eta =\left(\begin{array}{c} \sqrt{f_{\text{ex}}}\\ \sqrt{\frac{f_{\text{in}}}{N_{c}}}\\ \vdots \\ \sqrt{\frac{f_{\text{in}}}{N_{c}}} \end{array}\right),$$ (17)

which has an eigenvalue of$\lambda_{1}=0$. The last eigenvector forGis

$$\nu_{N}=\left(\begin{array}{c} -\sqrt{f_{\text{in}}}\\ \sqrt{\frac{f_{\text{ex}}}{N_{c}}}\\ \vdots \\ \sqrt{\frac{f_{\text{ex}}}{N_{c}}} \end{array}\right),$$ (18)

which has an eigenvalue of$\lambda_{N}=-R_{\text{in}}/f_{\text{ex}}$. Note that$\left|\nu_{1}\right|=\left|\nu_{N}\right|=1$and that$\nu_{1}\cdot \nu_{N}=0$. In addition, it is easy to show that

$$\sqrt{f_{\text{ex}}}\nu_{1}-\sqrt{f_{\text{in}}}\nu_{N}=\left(\begin{array}{c} 1\\ 0\\ \vdots \\ 0 \end{array}\right)\equiv u_{1}.$$ (19)

Therefore, theN−2dimensional subspace$U_{⊥}$that is orthogonal to the subspace spanned by$\nu_{1}$and$\nu_{N}$is orthogonal to$u_{1}$. By inspection, one then sees that any vector within$U_{⊥}$is an eigenvector ofGand has an eigenvalue equal to$-R_{\text{in}}$. It follows that any orthonormal basis for$U_{⊥}$completes the set ofNeigenvectors forG. ThatGalways asN−2degenerate eigenvalues greatly simplifies the analysis of this model.

By applyingEquations 4,6,14,17, and18, one obtains

$$K\left(t\right)=\left(K_{0}-\kappa_{N}\right)ϒ\left(R_{\text{in}}t\right)+\kappa_{N}ϒ\left(\frac{R_{\text{in}}t}{f_{\text{ex}}}\right),$$ (20)

with

$$K_{0}=3f_{\text{ex}}{\left(\frac{D_{1}}{D}-1\right)}^{2}+\frac{3f_{\text{in}}}{N_{c}}\sum_{n=2}^{N}{\left(\frac{D_{n}}{D}-1\right)}^{2}$$ (21)

and

$$\kappa_{N}=\frac{3f_{\text{ex}}f_{\text{in}}}{D^{2}}{\left(D_{1}-\frac{1}{N_{c}}\sum_{n=2}^{N}D_{n}\right)}^{2}.$$ (22)

FromEquation 3, we also find

$$R_{\text{KM}}=R_{\text{in}}\left(1+\frac{f_{\text{in}}\kappa_{N}}{f_{\text{ex}}K_{0}}\right),$$ (23)

which gives the connection between$R_{\text{KM}}$and the model parameters.Figure 1Bis a plot based onEquation 23of$R_{\text{KM}}/R_{\text{in}}$as a function$\kappa_{N}/K_{0}$for several values of$f_{\text{ex}}$.

In the special case that all the neurites are oriented in the same direction, the intracompartmental neurite diffusivities are identical, and our model becomes effectively a two-compartment KM. Then, we have$\kappa_{N}=K_{0}$and$R_{\text{KM}}={\hat{R}}_{\text{KM}}$. Another notable case is when$D_{1}$is equal to the average of the neurites’ intracompartmental diffusvities so that$\kappa_{N}=0$. As a consequence,Equation 20has only a single term, and the kurtosis behaves just like that of a two-compartment KM with the enhanced lower bound being exact once again. However, in this second instance, the KM still has, if$N_{c}>1$, more than two distinct compartments, and the full dMRI signal deviates, in general, from the two-compartment form.

### 2.6 Rotational invariants

Equation 14is the fundamental result that underlies our approach for determining the lower bounds$R_{\text{KM}}^{*}$and${\hat{R}}_{\text{KM}}$. It applies to any chosen diffusion direction. Moreover, it continues to be valid for linear combinations of the kurtosis from different directions. Hence, the mean kurtosis (MK), which is simply the kurtosis averaged over all directions, also satisfies this equation, and we are free to equate our parameterKwith MK. The advantage of this is that MK is a rotational invariant, as are the lower bounds$R_{\text{KM}}^{*}$and${\hat{R}}_{\text{KM}}$derived from MK, which would not necessarily be the case if only a single direction were used. In this study, we therefore setKequal MK when applyingEquation 14to experimental data as rotational invariants are generally of primary interest. Similarly, we identify the parameterDwith the mean diffusivity (MD) when usingEquation 7to calculate the diffusion elasticityξfrom experimental data.

## 3 Methods

### 3.1 Accuracy of lower bound for thin cylindrical neurite model

The accuracies of$R_{\text{KM}}^{*}$and${\hat{R}}_{\text{KM}}$as estimates for$R_{\text{KM}}$were quantified by the ratios$R_{\text{KM}}^{*}/R_{\text{KM}}$and${\hat{R}}_{\text{KM}}/R_{\text{KM}}$, which are guaranteed to be ≤1 byEquation 9. We calculated these accuracies for the thin cylindrical neurite model as functions of$R_{\text{KM}}^{*}t^{*}$since this is a convenient parameter to measure experimentally. While$R_{\text{KM}}^{*}t^{*}$can in principle be as large as 3, we only considered values up to 2 as that covers the range of greatest practical relevance.$R_{\text{KM}}^{*}$was obtained by applyingEquations 8to20,${\hat{R}}_{\text{KM}}$was determined fromEquation 9, and$R_{\text{KM}}$was found fromEquation 23. The accuracies were then calculated for$f_{\text{ex}}=$0.2, 0.4, 0.6, 0.8 and$\kappa_{N}/K_{0}=$ 0, 0.2, 0.4, 0.6, 0.8, 1.0. This broad range of parameters was chosen for the sake of completeness rather than a specific biological motivation. Certainly, an extra-neurite water fraction of$f_{\text{ex}}=$ 0.8 would not correspond to healthy brain, but might be relevant for pathological tissue due to stroke or other severe injury.

### 3.2 Animals

A total of 6 eight-month-old female mice were used in this study under a protocol approved by the Institutional Animal Care and Use Committee of the Medical University of South Carolina (Public Health Service Animal Welfare Assurance D16-00268 [A3428-01]). Of these, 3 were normal control (NC) mice and 3 were transgenic (TG) mice. The NC mice (C57BL/6J) were acquired from The Jackson Laboratories (Bar Harbor, ME, United States). The TG mice were obtained from the Mutant Mouse Resource and Research Center (MMRRC), an NIH-funded strain repository at The Jackson Laboratory, and were the 3xTg-AD model (B6;129 Tg(APPSwe,tauP301L)1LfaPsen1tm1Mpm/Mmjax, RRID:MMRRC_034830-JAX), donated to the MMRRC by Frank Laferla, PhD, University of California, Irvine. This TG model develops the main features of Alzheimer’s pathology, including amyloid-β plaques and neurofibrillary tangles (Javonillo et al., 2022;Oddo et al., 2003). All efforts were made to minimize the suffering of animals used in this study.

### 3.3 Image acquisition

Imaging was performed on a 7 T Bruker Biospec 70/30 MRI scanner running Paravision Version 5.1. Mice were anesthetized with isoflurane, and body temperature maintained with warm air. To reduce motion artifacts, mice were restrained by a holder and tooth bar. Diffusional kurtosis imaging (DKI;Jensen & Helpern, 2010;Jensen et al., 2005) data were acquired using a two-shot EPI sequence with the imaging parameters: TR = 3,750 ms, TE = 47 ms, slice thickness = 0.7 mm, field of view = 20 × 20 mm^2^, matrix = 128 × 128, gradient pulse duration δ = 5 ms,*b*-values = 0, 500, 1,000, 1,500, 2,000 s/mm^2^, diffusion times Δ = 18, 22, 26, 30 ms, and number of diffusion directions = 30. For each diffusion time, we obtained 10 volumes with*b*-value = 0.

### 3.4 DKI analysis

Parametric maps of the mean diffusivity (MD), mean kurtosis (MK), and fractional anisotropy (FA) were generated using Diffusional Kurtosis Estimator (Tabesh et al., 2011;https://www.nitrc.org/projects/dke/). Regions of interest (ROIs) within the DH and CX were defined on the FA maps of a single anatomical slice for each animal by an experienced neuropathologist (M.F.F.). Data from both hemispheres were combined. Examples of the ROIs are shown inFigure 2. The mean ROI values for MD and MK were extracted from the maps with ImageJ (Schneider et al., 2012;https://imagej.net/ij/). The diffusion elasticityξwas found from the slope ofD(set equal to MD) versus the diffusion timeton a log-log plot as determined by a linear least squares fit. The lower bound$R_{\text{KM}}^{*}$was obtained from the slope ofK(set equal to MK) versus the diffusion timeton a semi-log plot. Finally,${\hat{R}}_{\text{KM}}$was calculated from theEquations 9and10along with the measured values for$R_{\text{KM}}^{*}t^{*}$. In our analysis,$t^{*}$was set equal to 24 ms, which is the average of the 4 diffusion times.

**Fig. 2.**
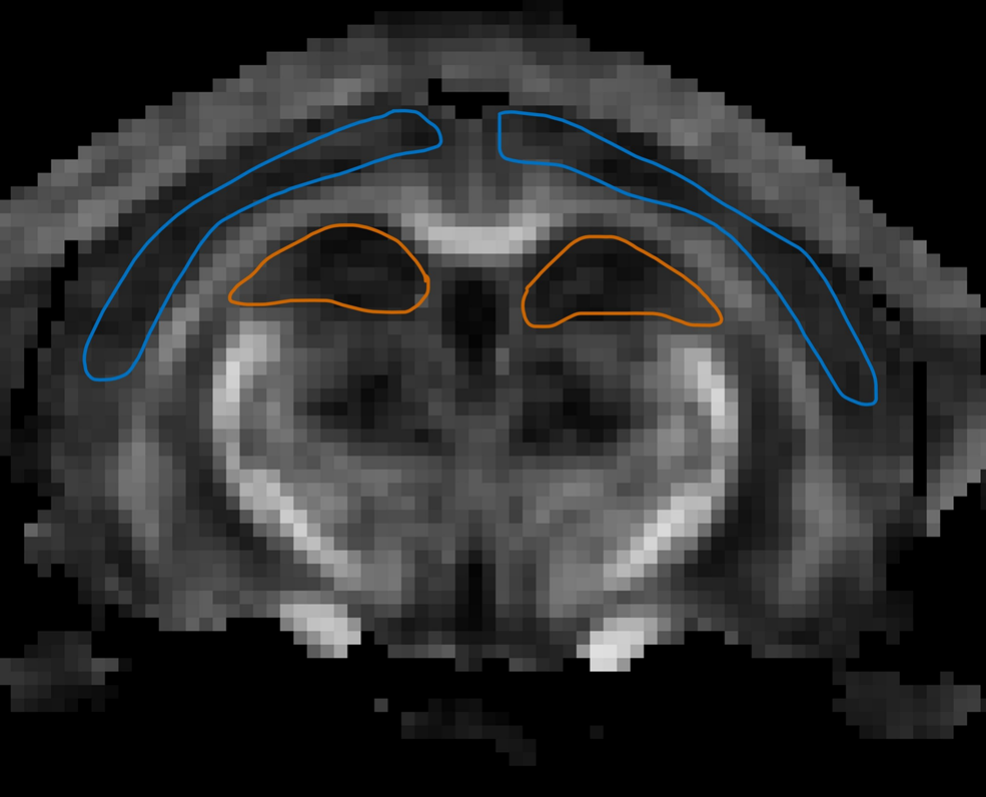
Fractional anisotropy (FA) map from one animal illustrating the two regions of interest (ROIs) used in this study for the dorsal hippocampus (outlined in orange) and cortex (outlined in blue). For each animal, data were pooled from both hemispheres of the brain within a single coronal slice.

### 3.5 Histology

Since myelination can affect water exchange rates for axons (Brusini et al., 2019), 25 µm histological sections were prepared for 1 eight-month-old TG mouse with staining for myelin basic protein (MBP; ab40390, Abcam, Cambridge, MA, United States). While not one of the mice that were scanned, this TG mouse was of the same age and strain. The mouse brain was fixed overnight and processed by Neuroscience Associates (Knoxville, TN, United States). A single histological section was selected corresponding to a similar brain region as used in the DKI analysis. This section was digitized following a previously described protocol (Falangola et al., 2023). Using ImageJ, ROIs for the DH and CX were defined, and the mean intensities for each ROI were measured. The degree of MBP immunoreactivity was quantified in DH and CX by using the optical density (OD) calculated as the logarithm of the ratio of the maximum image intensity for the section to the mean intensity of the ROI (Oberholzer et al., 1996).

## 4 Results

### 4.1 Accuracy of lower bounds for thin cylindrical neurite model

The accuracy of$R_{\text{KM}}^{*}$as an estimate of$R_{\text{KM}}$is given byFigure 3. The accuracies for$\kappa_{N}/K_{0}=$0 and$\kappa_{N}/K_{0}=$ 1 are identical and independent of$f_{\text{ex}}$. For other values of$\kappa_{N}/K_{0}$with fixed$R_{\text{KM}}^{*}t^{*}$and$f_{\text{ex}}$, the accuracies are always lower. The accuracies’ sensitivity to$\kappa_{N}/K_{0}$decrease as$f_{\text{ex}}$is increased. Increasing$R_{\text{KM}}^{*}t^{*}$reduces the accuracies, and they always approach 100% in the limit$R_{\text{KM}}^{*}t^{*}\to 0$. For$2<R_{\text{KM}}^{*}t^{*}<3$(not shown), they continue to decrease and approach zero as$R_{\text{KM}}^{*}t^{*}\to 3$. The practical utility of$R_{\text{KM}}^{*}$as an estimate for$R_{\text{KM}}$is then limited for$R_{\text{KM}}^{*}t^{*}>2$.

**Fig. 3.**
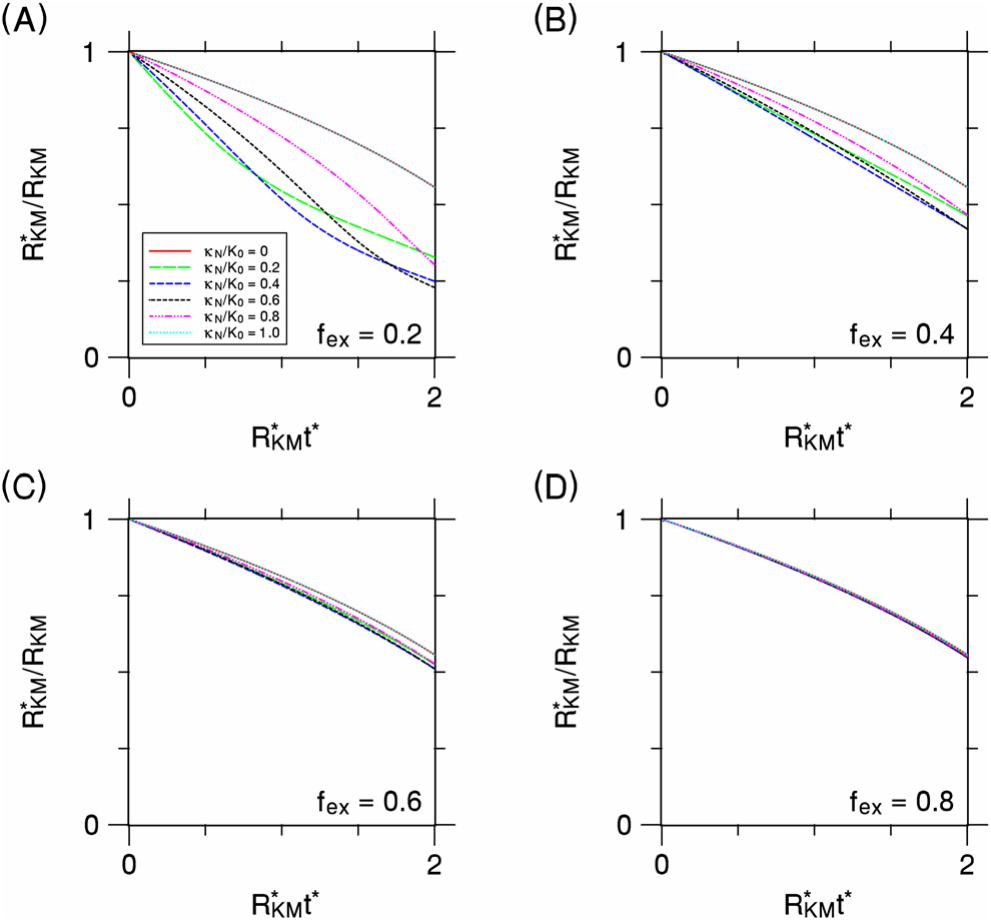
Accuracy of the lower bound$R_{KM}^{*}$expressed as the ratio$R_{KM}^{*}/R_{KM}$as a function of$R_{KM}^{*}t^{*}$in the thin cylindrical neurite model (A-D). For the cases considered, the accuracy is better than 73% for$R_{KM}^{*}t^{*}\leq 0.5$and better than 51% for$R_{KM}^{*}t^{*}\leq 1.0$. The accuracy becomes less sensitive to$\kappa_{N}/K_{0}$as$f_{\text{ex}}$is increased. The accuracies with$\kappa_{N}/K_{0}=0$and$\kappa_{N}/K_{0}=1$are identical and independent of$f_{\text{ex}}$.

**Fig. 4.**
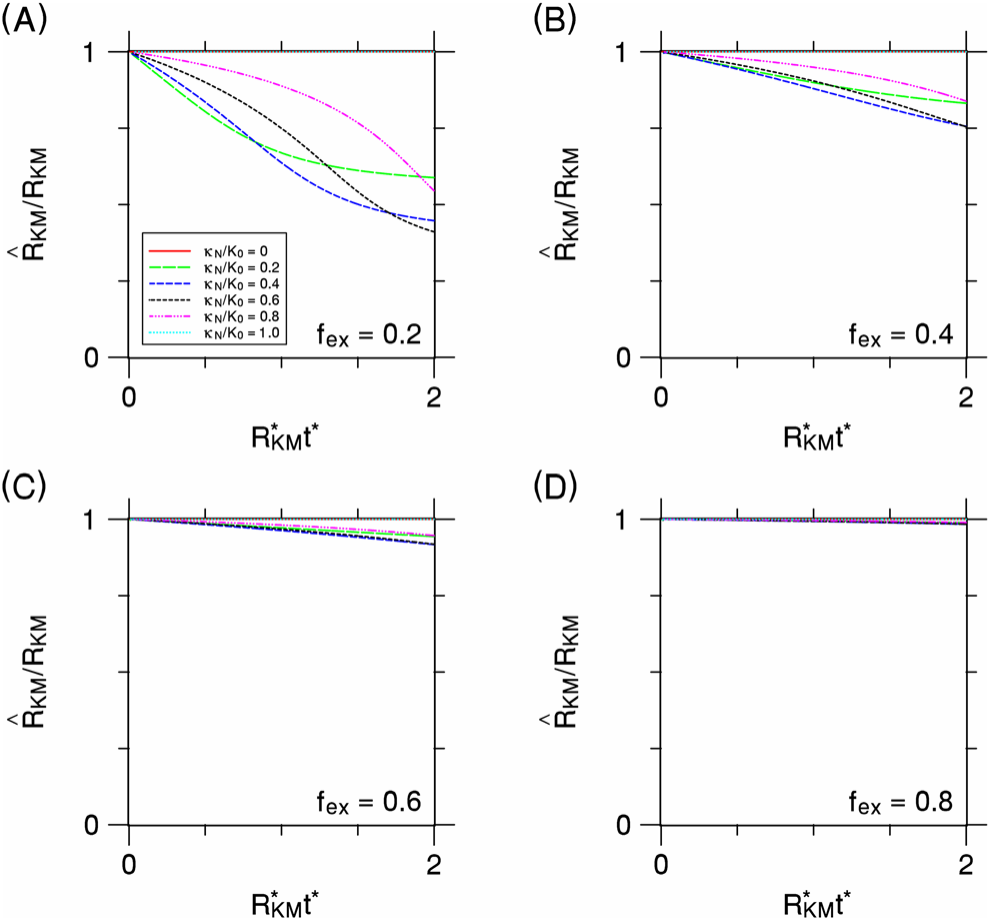
Accuracy of the lower bound${\hat{R}}_{KM}$expressed as the ratio${\hat{R}}_{KM}/R_{KM}$as a function of$R_{KM}^{*}t^{*}$in the thin cylindrical neurite model (A-D). For the cases considered, the accuracy is better than 80% for$R_{KM}^{*}t^{*}\leq$0.5 and better than 63% for$R_{KM}^{*}t^{*}\leq$1.0. The accuracies are 100% with$\kappa_{N}/K_{0}=$0 and$\kappa_{N}/K_{0}=$ 1 for all values of$f_{ex}$. The lower bound${\hat{R}}_{KM}$is always more accurate than$R_{KM}^{*}$.

Figure 4shows the accuracy of${\hat{R}}_{\text{KM}}$as an estimate of$R_{\text{KM}}$for the same range of model parameters as inFigure 3. Comparison withFigure 3illustrates the improved accuracy provided by${\hat{R}}_{\text{KM}}$relative to$R_{\text{KM}}^{*}$. The improvement is 9.6% for$R_{\text{KM}}^{*}t^{*}=$ 0.5, 23.0% for$R_{\text{KM}}^{*}t^{*}=$ 1.0, 43.3% for$R_{\text{KM}}^{*}t^{*}=$ 1.5, and 79.7% for$R_{\text{KM}}^{*}t^{*}=$ 2.0. For$\kappa_{N}/K_{0}$= 0 and$\kappa_{N}/K_{0}=$1, we have${\hat{R}}_{\text{KM}}=$$R_{\text{KM}}$, yielding an accuracy of 100%. As$R_{\text{KM}}^{*}t^{*}\to 3$, the accuracy of${\hat{R}}_{\text{KM}}$for this model approaches a constant (not shown), but the enhancement factor$E_{f}$diverges (Fig. 1A). Consequently, any experimental errors would be amplified, resulting in low precision estimates of$R_{\text{KM}}$for large values of$R_{\text{KM}}^{*}t^{*}$.

### 4.2 DKI analysis

The diffusivity as a function of the diffusion time is plotted inFigure 5, with the solid lines indicating linear least-squares fits. The diffusivity changes little over the considered range oft= 18 to 30 ms and is similar across regions and groups. Some of the scatter in the measurements from individual animals (data points) may be due to signal noise and imaging artifacts. On physical grounds, the true diffusivity should not increase as the diffusion time is lengthened.Figure 6gives a log-log plot of the same data shown inFigure 5. The slopes of the linear fits provide estimates of the diffusion elasticityξ. For both regions and both groups,|ξ|<0.05. Taking into account the statistical uncertainties (listed inFig. 6andTable 1), the measuredξvalues are consistent with the KM prediction ofξ=0.

**Fig. 5.**
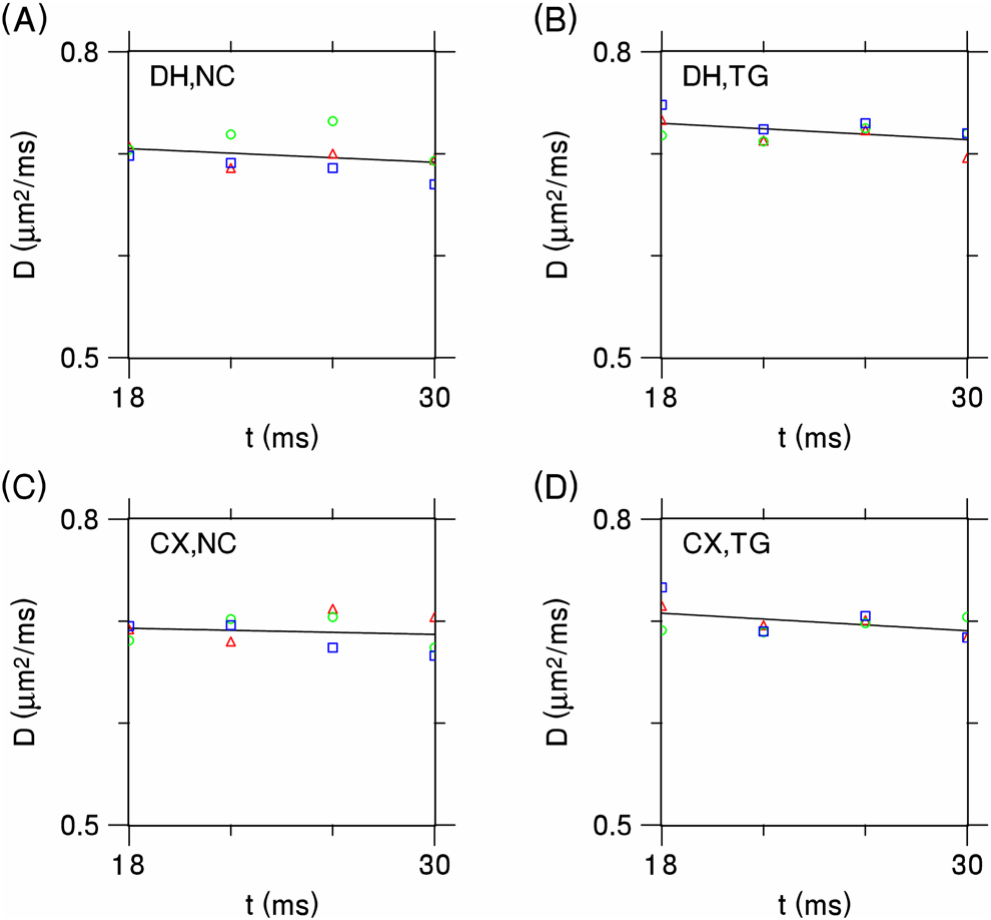
The mean diffusivity as a function of diffusion time for the dorsal hippocampus (DH) and the cortex (CX). Data from the normal control (NC) mice are shown in panels (A) and (C), while data from the transgenic (TG) mice are shown in panels (B) and (D). The data points show mean values for individual animals, with distinct symbols distinguishing animals within a group. Linear least-squares fits (solid lines) indicate that the diffusivity varies little over the time range considered and is similar across regions and groups.

**Fig. 6.**
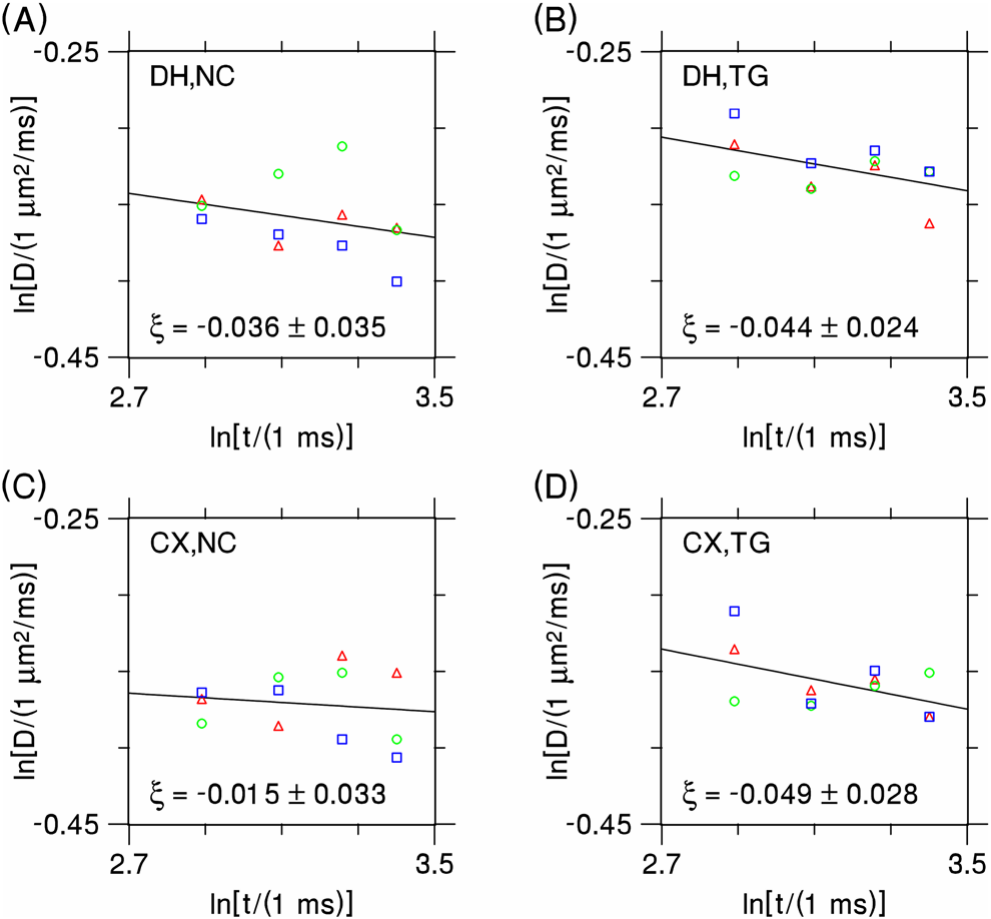
Log-log plot of the same data shown inFigure 5(A-D). The slopes of the linear fits (solid lines) give the diffusion elasticityξ, which provides a quantitative criterion for assessing the strength of the diffusivity’s time dependence. In all cases, the elasticity has a magnitude much less than one implying that the mean diffusivity can be considered to be approximately constant for diffusion times between 18 and 30 ms in consistency with the KM.

**Table 1.** Exchange-related parameters estimated in mouse dorsal hippocampus (DH) and cortex (CX).

Region	Group	ξ	$R_{KM}^{*}$ (s ^-1^ )	$R_{KM}^{*}t^{*}$	$E_{f}$	${\hat{R}}_{KM}$ (s ^-1^ )
DH	NC	-0.036 ± 0.035	40.7 ± 10.4	0.98 ± 0.25	1.22 ± 0.08	49.8 ± 15.9
DH	TG	-0.044 ± 0.024	35.9 ± 11.1	0.86 ± 0.27	1.18 ± 0.07	42.6 ± 16.2
DH	All	-0.040 ± 0.027	38.3 ± 7.4	0.92 ± 0.18	1.20 ± 0.05	46.1 ± 11.0
CX	NC	-0.015 ± 0.033	15.7 ± 10.7	0.38 ± 0.26	1.07 ± 0.05	16.8 ± 12.3
CX	TG	-0.049 ± 0.028	22.0 ± 10.1	0.53 ± 0.24	1.10 ± 0.05	24.3 ± 12.4
CX	All	-0.032 ± 0.022	18.9 ± 7.2	0.45 ± 0.17	1.09 ± 0.04	20.5 ± 8.5

The All group is for data pooled from the normal control (NC) and transgenic (TG) mice.

The mean kurtosis values for each animal are plotted versus time inFigure 7. The fit lines indicate that the kurtosis decreases between 18 and 30 ms.Figure 8is a semi-log plot of the same data. The slopes of the lines inFigure 8correspond to the logarithmic derivative of the kurtosis with respect to time and, by applyingEquation 8, yield estimates for$R_{\text{KM}}^{*}$.

**Fig. 7.**
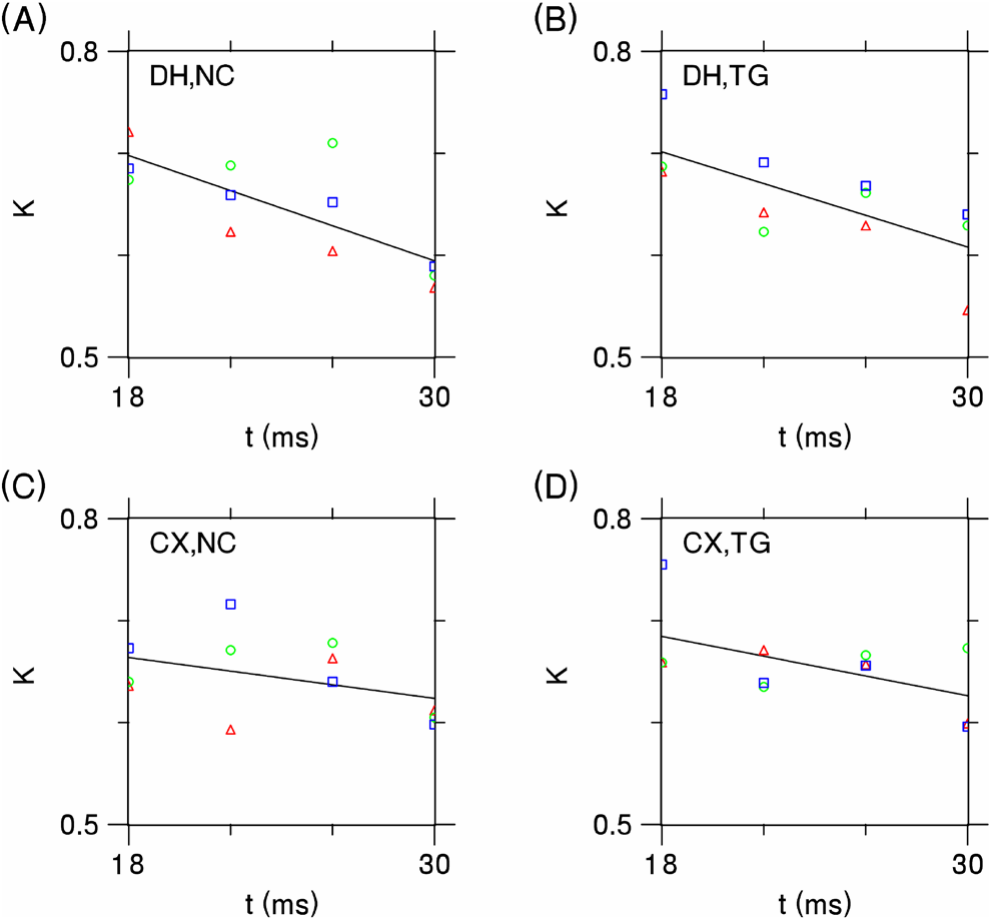
The mean kurtosis as a function of diffusion time for the two ROIs. The data from the NC mice are shown in panels (A) and (C), and the data from the TG mice are shown in panels (B) and (D). Linear fits (solid lines) indicate that the kurtosis decreases with increasing diffusion time.

**Fig. 8.**
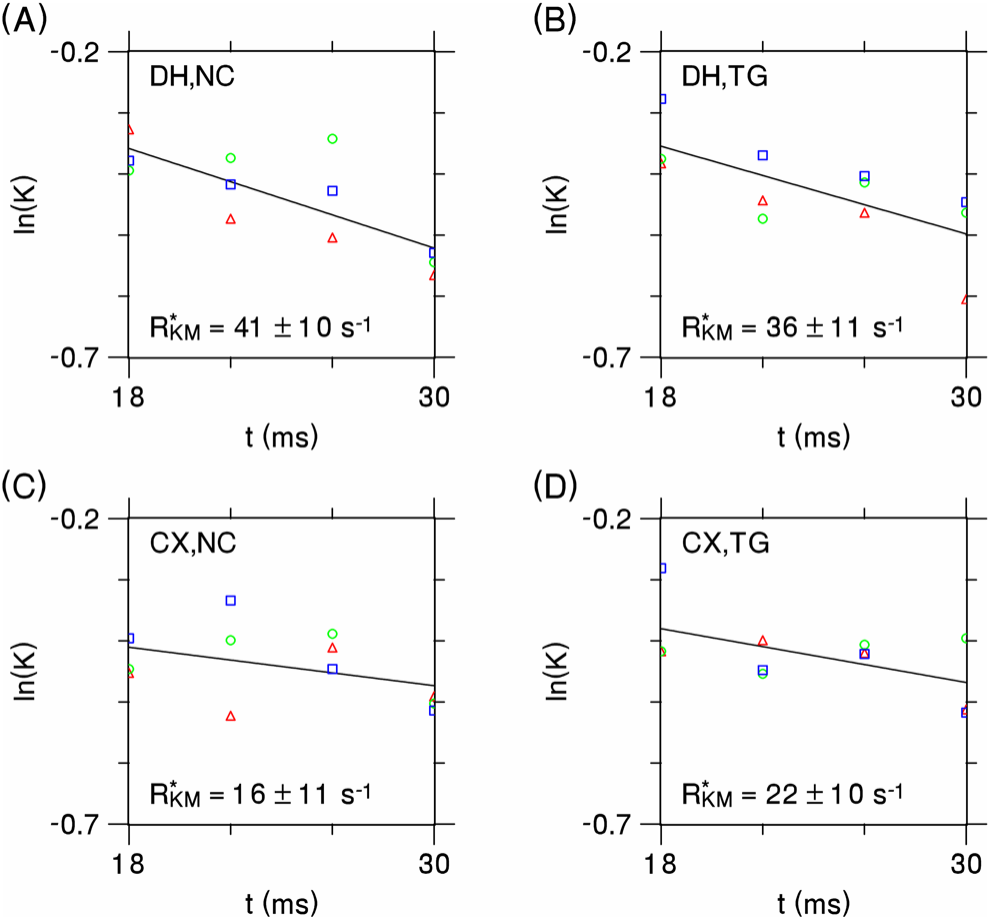
Semi-log plot of the same data shown inFigure 7(A-D). The slopes of the linear fits (solid lines) times -3 give the lower bound$R_{KM}^{*}$for the mean KM exchange rate. The values for$R_{KM}^{*}$are similar for NC and TG mice.

Our results are summarized inTable 1, which includes values for the individual groups as well as for the “All” group in which data from NC and TG animals are combined. The pooling of the NC and TG data is justified since there are no statistically significant differences between these two groups for any of the parameters considered. For the All group, the difference between$R_{\text{KM}}^{*}$in DH and$R_{\text{KM}}^{*}$in CX is 19.4 ± 10.3 s^-1^. This is not significantly different from zero, but the*p*-value for a Z-test (*p*= 0.06) is close to being significant when the alpha level is set to 0.05. The enhanced lower bound${\hat{R}}_{\text{KM}}$is about 20% larger than$R_{\text{KM}}^{*}$in DH and about 9% larger in CX.

### 4.3 Histology

The brain section stained with MBP is shown inFigure 9. Much stronger MBP immunoreactivity is found in CX relative to DH. This is also reflected in the larger mean OD in CX of 22.6 compared to 6.6 in DH, implying that the CX myelin content is several times higher.

**Fig. 9.**
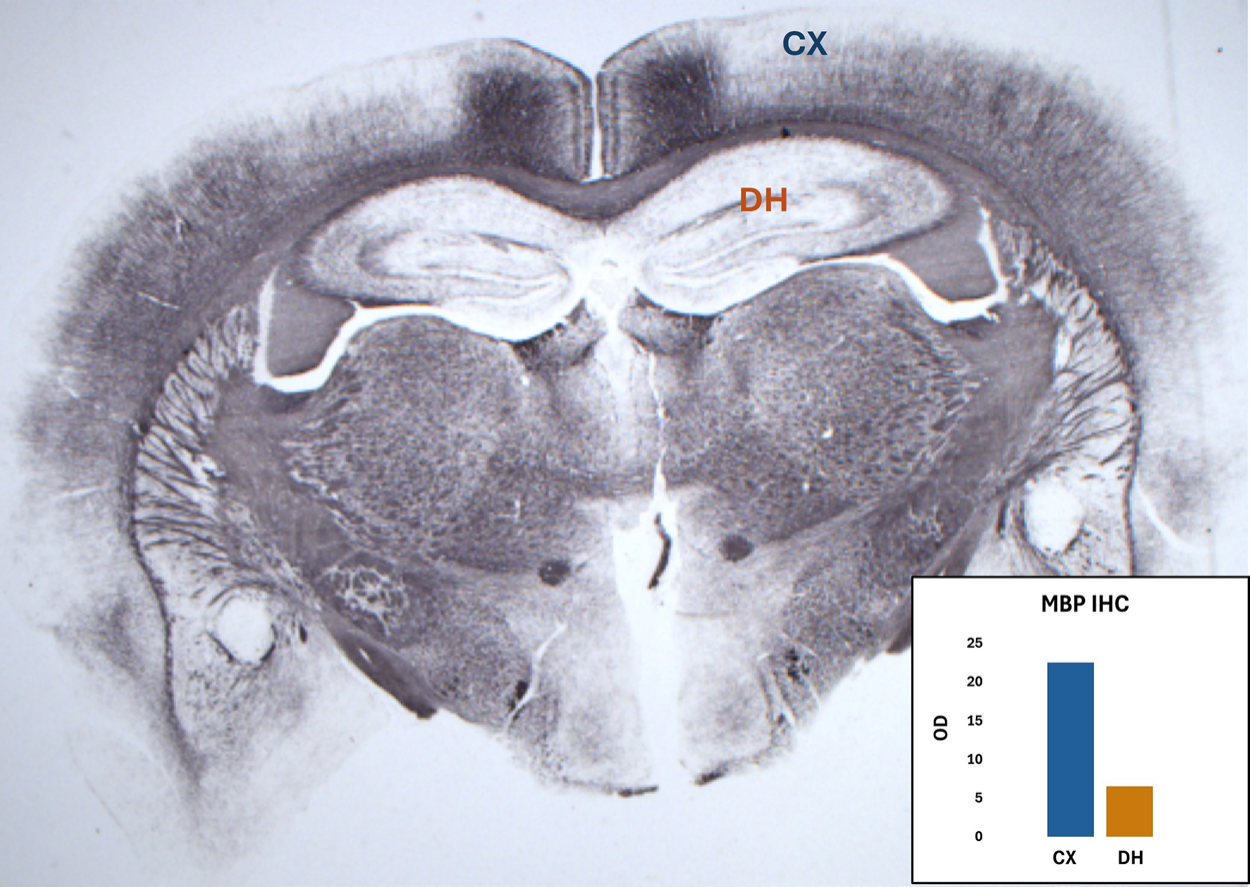
Myelin basic protein (MBP) stain (1.25× magnification) of a TG mouse obtained with immunohistochemistry (IHC). The optical density (OD) is several times higher in CX compared to the DH indicating a substantially greater degree of myelination in CX. This difference could contribute to the observed higher$R_{KM}^{*}$values for DH since water exchange is expected to be slower for myelinated axons.

## 5 Discussion

A comprehensive characterization of the myriad water exchange processes occurring within brain tissue is likely beyond the capabilities of diffusion MRI. Therefore, a more realistic goal is establishing summary statistics that capture salient aspects of water exchange and are amenable to experimental measurement. Ideally, such summary statistics would not be reliant on detailed modeling assumptions so they are applicable across brain regions and in the presence of pathology. Here, we have investigated the mean KM water exchange rate,$R_{KM}$, as one proposed summary statistic of this type.

In prior work (Jensen, 2024), it is shown how to determine a lower bound on$R_{\text{KM}}$from the slope of the logarithmic derivative of the diffusional kurtosis with respect to time. Here we have improved upon this bound,$R_{\text{KM}}^{*}$, by deriving an enhancement factor$E_{f}$that can be calculated from the product$R_{\text{KM}}^{*}t^{*}$, where$t^{*}$is the diffusion time used to find$R_{\text{KM}}^{*}$. The stronger bound is then${\hat{R}}_{\text{KM}}=E_{f}R_{\text{KM}}^{*}$, which is more than 10% larger than$R_{\text{KM}}^{*}$for$R_{\text{KM}}^{*}t^{*}\geq 0.52$, and more than 22% larger for$R_{\text{KM}}^{*}t^{*}\geq 1.0$. Since obtaining${\hat{R}}_{\text{KM}}$does not require additional measurements, it should be preferred over$R_{\text{KM}}^{*}$as an estimate for$R_{\text{KM}}$.

In order to assess the accuracy of$R_{\text{KM}}^{*}$and${\hat{R}}_{\text{KM}}$, we have considered a specific KM comprising an arbitrary number of thin cylindrical neurites. All the neurites are identical but may have different spatial orientations. While highly simplified, this model, nonetheless, captures some basic features of brain microstructure and is similar to the well-known “Standard Model” of neuronal tissue (Novikov et al., 2019) although the extra-neurite space is treated differently. For the thin cylindrical neurite model, we have derived an exact formula giving the time dependence of the kurtosis and compared$R_{\text{KM}}$to the approximations of$R_{\text{KM}}^{*}$and${\hat{R}}_{\text{KM}}$. We find the accuracy to depend significantly on the water fraction for the extra-neurite space$f_{\text{ex}}$. The accuracy of$R_{\text{KM}}^{*}$decreases with increasing$R_{\text{KM}}^{*}t^{*}$, and becomes poor for$R_{\text{KM}}^{*}t^{*}>2$. For${\hat{R}}_{\text{KM}}$, the accuracy can be substantially higher, but the enhancement factor diverges as$R_{\text{KM}}^{*}t^{*}\to 3$, thereby greatly amplifying any experimental errors. In practice, the two lower bounds would usually be most useful when$R_{\text{KM}}^{*}t^{*}<2$.

Based on histology, neurites have been reported to occupy about 60% of the neuropil volume (Chklovskii et al., 2002), which implies$f_{\text{ex}}\approx$0.4. Assuming this value, the accuracy of$R_{KM}^{*}$for the thin cylindrical neurite model is (depending of$\kappa_{N}$) 86% to 91% for$R_{\text{KM}}^{*}t^{*}=$0.5 and 71% to 81% for$R_{\text{KM}}^{*}t^{*}=$ 1.0. The corresponding accuracies for${\hat{R}}_{\text{KM}}$are 94% to 100% and 88% to 100%. Since histological estimates of volume fractions sometime underestimate the amount of extracellular space (Cragg, 1979), the in vivo extra-neurite space (comprising both the extracellular space and glia) might have a volume fraction as large as 0.5, assuming an extracellular fraction of 0.2 (Nicholson & Hrabětová, 2017). The accuracies of$R_{KM}^{*}$then become 88% to 91% for$R_{\text{KM}}^{*}t^{*}=$0.5 and 76% to 81% for$R_{\text{KM}}^{*}t^{*}=$1.0 while for${\hat{R}}_{\text{KM}}$they would be 97% to 100% and 93% to 100%. These results suggest what may be reasonably expected for the accuracy of measurements performed in gray matter. Nevertheless, it should be noted that our model is unrealistic in not containing cell bodies and disregarding water exchange for glia.

For our measurements in CX, we find$R_{\text{KM}}^{*}=$18.9 ± 7.2 s^-1^and${\hat{R}}_{\text{KM}}=$20.5 ± 8.5 s^-1^when data from the NC and TG mice are combined. These are similar to the value of$R_{\text{KM}}^{*}=$26 ± 1 s^-1^previously estimated in CX of 6 to 8 weeks old rat brain (Jensen, 2024), based on data fromPyatigorskaya et al. (2014). They can also be compared to the CX exchange time of 41 ± 18 ms obtained byJelescu et al. (2022)from fitting the two-compartment KM kurtosis to rat brain data. For this model,$R_{KM}$is simply equal to the inverse exchange time, leading to$R_{\text{KM}}=$24 ± 11 s^-1^.Jelescu et al. (2022)also estimate an exchange time of 28 ± 15 ms in hippocampus, which corresponds to$R_{\text{KM}}=$36 ± 19 s^-1^. This is close to our DH values of$R_{\text{KM}}^{*}=$38.3 ± 7.4 s^-1^and${\hat{R}}_{\text{KM}}=$ 46.1 ± 11.0 s^-1^. The All group experimental values for$R_{\text{KM}}^{*}t*$are 0.92 for DH and 0.45 for CX. Our numerical calculations using the thin cylindrical neurite model with$f_{ex}=0.4$then indicate an accuracy for${\hat{R}}_{\text{KM}}$as an estimate of$R_{\text{KM}}$to be 88% or better in DH and 94% or better in CX.

Although not quite statistically significant, we find$R_{\text{KM}}^{*}$in DH to be over twice that in CX for the pooled data. Regional variations in$R_{\text{KM}}^{*}$could be caused by differences in cell density, morphology, and/or membrane permeability that affect water exchange. One factor that might contribute to a lower$R_{\text{KM}}^{*}$in CX is its greater degree of myelination, as demonstrated by the MBP stain shown inFigure 9. The water permeability of myelinated axons is presumably lower than for dendrites and unmyelinated axons (Brusini et al., 2019), and they would therefore tend to decrease exchange rates.

The time dependence of the kurtosis has also been measured in fixed brain cortex for mouse (Aggarwal et al., 2020) and rat (Olesen et al., 2022). For the mouse data, we previously estimated$R_{\text{KM}}^{*}=76\pm 10$s^-1^(Jensen, 2024), and a similar analysis applied to the rat data leads to$R_{\text{KM}}^{*}=114\pm 5$s^-1^. These are substantially higher than the in vivo values discussed above. This discrepancy is not surprising considering the profound microstructural changes associated with the fixation process, including a decrease in the extracellular space (Cragg, 1979) and alterations in diffusivity (Wang et al., 2018). Indeed,Equation 23suggests that both of these factors could potentially affect$R_{\text{KM}}$.

Our interpretations of$R_{\text{KM}}^{*}$and${\hat{R}}_{\text{KM}}$are predicated on an assumption that the KM provides a good description of the diffusion dynamics for the range of diffusion times considered. The weak dependence of the diffusivity on diffusion time supports this assumption. In particular, the diffusion elasticities all have magnitudes that are below 0.05 and not statistically different from the KM prediction of zero. However, the most common types of neuron in mouse DH and CT, pyramidal cells, have soma diameters of 10 to 16 µm (Benavides-Piccione et al., 2020;Gilman et al., 2017), making this compartment too large to be fully explored by water molecules over the diffusion times used in our experiment. Hence, one of the conditions required for the validity of the KM may not be strictly satisfied. Any effects of this on our values for$R_{\text{KM}}^{*}$and${\hat{R}}_{\text{KM}}$are, nevertheless, likely to be modest since the volume fractions occupied by neuronal somas are small. These volume fractions can be estimated from the neuron cell densities reported in the mouse brain cell atlas from the Blue Brain Project of 1.25 × 10^5^/mm^3^for the “hippocampal region” and 1.02 × 10^5^/mm^3^for CX (Erö et al., 2018). Assuming spherical somas with a diameter of 13 µm, we then find volume fractions of 14% for DH and 12% for CX.

An advantage of$R_{KM}$is that it is well defined for all KMs regardless of the number of compartments or specific values of the model parameters. Thus, for any particular model, one may derive explicit expressions for$R_{KM}$, such asEquation 23in the case of the thin cylindrical neurite model. A similar formula can be demonstrated for the neurite exchange imaging (NEXI) model (Jelescu et al., 2022), which is based on an orientationally-averaged two-compartment anisotropic KM. This generality allows for direct comparisons of water exchange predictions across distinct KM variants. Moreover,$R_{\text{KM}}^{*}$has a purely empirical definition as -3 times the minimum of the logarithmic derivative of the kurtosis with respect to time (seeEquation 8). Therefore, it can be calculated whenever data for the kurtosis as a function of time are available and can serve as a convenient indicator of the magnitude of water exchange. However, it should be remembered that measured values of$R_{\text{KM}}^{*}$will typically depend on the range of diffusion times employed. In most cases, shorter diffusion times are preferred as these yield tighter bounds on$R_{\text{KM}}$.

Since they are defined in terms of the logarithmic derivative ofK(t)at a single time point,$R_{\text{KM}}^{*}$and${\hat{R}}_{\text{KM}}$can be estimated in practice from a relatively narrow range of diffusion times, as illustrated by the experimental results presented here, provided these are sufficient for estimating the slope ofln[K(t)]. This differs from the more usual approach of fitting a theoretically predictedK(t)for a specific KM to experimental data over a broader range of diffusion times (Jelescu et al., 2022;Lee et al., 2020;Mougel et al., 2024;Uhl et al., 2024;Zhang et al., 2021). By only requiring a short span of times, experimental protocols for the lower bounds are simpler and potentially more efficient. As demonstrated in our prior work by numerical calculations, the lower bounds can, at least in some cases, give more accurate estimates of$R_{\text{KM}}$than a conventional fit even when less data is utilized (Jensen, 2024).

A wide range of exchange rates for gray matter have been reported in the literature (Li et al., 2023). For example, an apparent exchange rate of 0.4 s^-1^has been observed for human brain using the filter exchange imaging method (Nilsson et al., 2013) while high*b*-value dMRI data have yielded exchange residence times of 2–7 ms (Lee et al., 2022), corresponding to an exchange rate in the range of 143–500 s^-1^. Several reasons can be suggested for this disparity. First, some variability could simply be due to actual physical differences in the samples, as discussed above for fixed versus in vivo brain tissue. Second, exchange rate measurements are technically challenging, with significant systematic bias being possible depending on the method utilized. Third, the models fit to the data may not always be adequate to describe the intricacy of brain microstructure. As a consequence, the precise physical meaning of estimated exchange rates can be unclear and differ across methods. The approach proposed here is intended to address this third source of inconsistency by being valid for any KM regardless of the number of compartments or other details. While still an idealization, the general multi-compartment KM encompasses a greater degree of complexity and relies on fewer assumptions than the more detailed models employed in some other approaches. It is hoped that this will help increase the reliability and interpretability of exchange rate estimates and facilitate a more meaningful comparison between experiments.

One limitation of this study is that each of the two groups has only 3 animals, which restricts our power for detecting group differences. Indeed, none are found here for$R_{\text{KM}}^{*}$and$R_{\text{KM}}$. However, a prior study of 8-month-old 3xTg-AD mice with a larger sample size (N = 28) has reported significant differences, compared to NC mice (N = 17), for several diffusion measures in the hippocampus (Falangola et al., 2021). Therefore, further work with greater numbers of animals would be of interest to examine whether water exchange also varies between these two groups. A second limitation is that the accuracies of$R_{\text{KM}}^{*}$and${\hat{R}}_{\text{KM}}$as estimates for$R_{\text{KM}}$have only been investigated for a simple analytic model. Additional tests of their accuracies using Monte Carlo stimulations for more realistic models of brain microstructure, similar to those of previous studies (Aggarwal et al., 2020;Fieremans et al., 2010), would be of value.

## 6 Conclusion

A general lower bound on the mean KM water exchange rate,$R_{\text{KM}}$, has been derived that can be estimated from the logarithmic derivative of the diffusional kurtosis with respect to time and improves upon a previously reported bound. A specific KM is analyzed that describes water diffusion through brain tissue with an arbitrary number of neurites idealized as thin cylinders. An analytic expression is given for the kurtosis, which is used to assess the accuracy of the lower bound as an estimate for$R_{\text{KM}}$over a range of model parameters. Application of the improved lower bound to experimental results for mouse brain indicates that$R_{\text{KM}}$is 46.1 ± 11.0 s^-1^or greater in DH and 20.5 ± 8.5 s^-1^or greater in CX.

## Data and Code Availability

Data are available upon reasonable request from a qualified investigator and completion of a use agreement with the corresponding author. The software used for the DKI analysis is publicly available athttps://www.nitrc.org/projects/dke/. The software used for ROI delineation is publicly available athttps://imagej.net/ij/.

## Author Contributions

J.H.J. conceived the project and wrote the paper. M.F.F. and J.V. performed the experiments and provided edits and comments for the paper. J.V. generated the DKI maps and extracted the parameter values. M.F.F. delineated the ROIs and supplied the histological analysis.

## Funding

This work was supported, in part, by grants from the National Institutes of Health (R01AG054159 and R01AG057602) and by the Litwin foundation.

## Declaration of Competing Interest

The authors have no competing financial interests to declare.

## Appendix: Derivation of Enhanced Lower Bound

In this appendix, we demonstrate that an enhanced lower bound,${\hat{R}}_{\text{KM}}$, for$R_{\text{KM}}$is given by$E_{f}R_{\text{KM}}^{*}$, where$E_{f}$is the enhancement factor defined byEquations 10–12. Our argument consists of three parts. First, we establish some basic mathematical facts related to the functionϒ(x), as defined byEquation 15, and the functionβ(x), as defined byEquation 12. Second, for an arbitrary KM as specified inEquation 14, the minimum value of$R_{\text{KM}}$is derived with fixed$R_{\text{KM}}^{*}$and$\tau_{n}$over all possible choices for$\kappa_{n}$. Finally, this initial solution is minimized with respect to$\tau_{n}$, while still keeping$R_{\text{KM}}^{*}$fixed. We find that the global minimum is precisely${\hat{R}}_{\text{KM}}$, which must then be a lower bound for any KM, as indicted byEquation 9.

### Mathematical Preliminaries

Our demonstration relies on several properties related to the functionsϒ(x)andβ(x). The first of these are, for finite*x*,

ϒ(x)>0,
(A. 1)

ϒ′(x)<0,
(A. 2)

$$\beta \left(x\right)=-\frac{3x{ϒ}^{\prime }\left(x\right)}{ϒ\left(x\right)}<3.$$
(A. 3)

β′(x)>0,
(A. 4)

and

β″(x)<0.
(A. 5)

The arguments ofϒ(x)andβ(x)are always assumed to be non-negative since negative values are not of physical interest in our context. The prime marks inEquations A.2–A.5denote derivatives.Equations A.1andA.2follow by inspection of the integral representation inEquation 15forϒ(x).Equation A.3may be verified using the closed form expression forϒ(x), also given byEquation 15, and then comparing withEquation 12.Equations A.4andA.5are readily checked numerically; formal proofs are straightforward, but these are lengthy and omitted here for the sake of brevity.

We will also require the two additional functions

$$h\left(x\right)\equiv h_{0}ϒ\left(x\right)+3x{ϒ}^{\prime }\left(x\right)=ϒ\left(x\right)\left[h_{0}-\beta \left(x\right)\right],$$
(A. 6)

where$h_{0}=h(0)$, and

$$w\left(x\right)\equiv \frac{h\left(x\right)}{x_{0}-x},$$
(A. 7)

where$x_{0}=\beta^{-1}\left(h_{0}\right)$. There is always a unique value for$x_{0}$provided$0<h_{0}<3$, which is the range of physical interest since$h_{0}$will eventually be set equal to$R_{KM}^{*}t^{*}.$These two functions have the properties

$$h\left(x\right)\{\begin{array}{c} >0,\;\;\;\;\;\text{if}\;x<x_{0},\\ =0,\;\;\;\;\;\text{if}\;x=x_{0},\\ <0,\;\;\;\;\;\text{if}\;x>x_{0}, \end{array}$$
(A. 8)

and

$$w^{\prime }(x)\leq 0.$$
(A. 9)

Equation A.8follows directly fromEquations A.1,A.4, andA.6. To demonstrateEquation A.9, we useEquations A.6andA.7to find

$$\frac{d}{dx}\left[\frac{w\left(x\right)}{ϒ\left(x\right)}\right]=\frac{1}{x_{0}-x}\left[\frac{h_{0}-\beta \left(x\right)}{x_{0}-x}-\beta '\left(x\right)\right].$$
(A. 10)

Equation A.5implies thatβ(x)is concave. As a consequence (Boyd & Vandenberghe, 2004),

$$\beta^{\prime }(x)\geq \frac{h_{0}-\beta \left(x\right)}{x_{0}-x},\;\;\;\;\text{if}\;x\leq x_{0},$$
(A. 11)

and

$$\beta^{\prime }\left(x\right)\leq \frac{h_{0}-\beta \left(x\right)}{x_{0}-x},\;\;\;\;\;\text{if}\;x\geq x_{0}.$$
(A. 12)

Applying this toEquation A.10yields

$$\frac{d}{dx}\left[\frac{w\left(x\right)}{ϒ\left(x\right)}\right]\leq 0.$$
(A. 13)

By using the chain rule for derivatives, one further finds

$$w^{\prime }\left(x\right)\leq \frac{w\left(x\right){ϒ}^{\prime }\left(x\right)}{ϒ\left(x\right)}.$$
(A. 14)

FromEquations A.7andA.8, it is apparent thatw(x)≥0for both$x>x_{0}$and$x<x_{0}$. For$x=x_{0}$, a direct calculation shows that$w\left(x_{0}\right)=ϒ\left(x_{0}\right)\beta \prime \left(x_{0}\right)$;Equations A.1andA.4then imply$\;w(x_{0})>0$.Equation A.9then follows fromEquations A.1,A.2, andA.14.

### Minimization Step 1

Now, we assume that$R_{\text{KM}}^{*}$is determined fromEquation 8at a given value oft=t*. Thus,

$$R_{\text{KM}}^{*}=-3\frac{K^{\prime }\left(t^{*}\right)}{K\left(t^{*}\right)}.$$
(A. 15)

FromEquation 14, one sees that this is equivalent to

$$R_{\text{KM}}^{*}=-3\sum_{n=2}^{N}\frac{\kappa_{n}}{\tau_{n}}ϒ\text{'}\left(\frac{t^{*}}{\tau_{n}}\right)/\sum_{n=2}^{N}\kappa_{n}ϒ\left(\frac{t^{*}}{\tau_{n}}\right).$$
(A. 16)

The initial optimization problem to be considered is then

$$\text{Minimize}:\;\;\;\;\;\;R_{\text{KM}}=\sum_{n=2}^{N}\frac{\kappa_{n}}{K_{0}\tau_{n}}$$
(A. 17)

subject to the constraints

$$R_{\text{KM}}^{*}\sum_{n=2}^{N}\kappa_{n}ϒ\left(\frac{t^{*}}{\tau_{n}}\right)+3\sum_{n=2}^{N}\frac{\kappa_{n}}{\tau_{n}}ϒ\text{'}\left(\frac{t^{*}}{\tau_{n}}\right)=0,$$
(A. 18)

$$K_{0}=\sum_{n=2}^{N}\kappa_{n},$$
(A. 19)

and

$$\kappa_{n}\geq 0,\;\;\;\;\;\;\;n=2,3,\ldots ,N.$$
(A. 20)

The constraint ofEquation A.19is the same asEquation 6. Without loss of generality, we take$K_{0}$to be fixed as our problem is invariant with respect to the rescaling$\kappa_{n}\to c\kappa_{n}$and$K_{0}\to cK_{0}$for an arbitrary constantc>0. TheN−1constraints ofEquation A.20follow fromEquation 4. We assume thatN>2since the case withN=2is trivial.

Equations A.17–A.20constitute a linear programming problem inN−1variables withN+1constraints, where we are regarding the$\tau_{n}$as fixed. The Fundamental Theorem of Linear Optimization then implies that the solution must occur for a set of$\kappa_{n}$values that satisfyN−1of the constraints as equalities (Press et al., 1992). Therefore, no more than two of the$\kappa_{n}$will be nonzero with the others being forced to vanish in order to satisfyEquation A.20. The solution to our optimization problem, thus, has the form

$$R_{\text{KM}}=\frac{\kappa_{a}}{K_{0}\tau_{a}}+\frac{\kappa_{b}}{K_{0}\tau_{b}}$$
(A. 21)

with$\kappa_{a}$and$\kappa_{b}$being determined from

$$\begin{array}{l} \kappa_{a}\left[R_{\text{KM}}^{*}ϒ\left(\frac{t^{*}}{\tau_{a}}\right)+\frac{3}{\tau_{a}}ϒ\prime \left(\frac{t^{*}}{\tau_{a}}\right)\right]\\ +\kappa_{b}\left[R_{\text{KM}}^{*}ϒ\left(\frac{t^{*}}{\tau_{b}}\right)+\frac{3}{\tau_{b}}ϒ\prime \left(\frac{t^{*}}{\tau_{b}}\right)\right]=0, \end{array}$$
(A. 22)

and

$$K_{0}=\kappa_{a}+\kappa_{b}.$$
(A. 23)

After solving for$\kappa_{a}$and$\kappa_{b}$, we obtain

$$R_{\text{KM}}t^{*}=\frac{x_{b}h\left(x_{a}\right)-x_{a}h\left(x_{b}\right)}{h\left(x_{a}\right)-h\left(x_{b}\right)},$$
(A. 24)

$$\kappa_{a}=\frac{K_{0}h\left(x_{b}\right)}{h\left(x_{b}\right)-h\left(x_{a}\right)},$$
(A. 25)

and

$$\kappa_{b}=\frac{K_{0}h\left(x_{a}\right)}{h\left(x_{a}\right)-h\left(x_{b}\right)},$$
(A. 26)

with$x_{a}\equiv t^{*}/\tau_{a}$,$x_{b}\equiv t^{*}/\tau_{b}$, and$h_{0}=R_{\text{KM}}^{*}t^{*}$. Since both$\kappa_{a}$and$\kappa_{b}$must be non-negative, we also have fromEquations A.25andA.26

$$0\leq \kappa_{a}\kappa_{b}=-\frac{K_{0}^{2}h\left(x_{a}\right)h\left(x_{b}\right)}{{\left[h\left(x_{a}\right)-h\left(x_{b}\right)\right]}^{2}},$$
(A. 27)

which implies 

$$h\left(x_{a}\right)h\left(x_{b}\right)\leq 0.$$
(A. 28)

Because ofEquation A.8, either$x_{a}$or$x_{b}\;$must be$\leq x_{0}$and either$x_{a}$or$x_{b}\;$must be$\geq x_{0}$. So, without loss of generality, we impose the condition

$$x_{a}\leq x_{0}\leq x_{b}.$$
(A. 29)

### Minimization Step 2

Equation A.24gives the minimum possible$R_{\text{KM}}$when the allowed exchange times are specified in advance. This optimum involves at most two distinct exchange times, which we have called$\tau_{a}$and$\tau_{b}$. To find the global minimum for$R_{KM}$, we must minimizeEquation A.24with respect to$\tau_{a}$and$\tau_{b}$, or equivalently with respect to$x_{a}$and$x_{b}$, while obeying the constraint ofEquation A.29.

FromEquations A.8andA.24, it is clear that if either$x_{a}=x_{0}$or$x_{b}=x_{0}$then$R_{KM}t^{*}=x_{0}$. This motivates rewritingEquation A.24as

$$R_{\text{KM}}t^{*}=x_{0}+\frac{\left(x_{0}-x_{a}\right)\left(x_{b}-x_{0}\right)}{h\left(x_{a}\right)-h\left(x_{b}\right)}\;\left[w\left(x_{a}\right)-w\left(x_{b}\right)\right].$$
(A. 30)

Because ofEquations A.8,A.9, andA.29, the second term on the right side ofEquation A.30can never be negative. Therefore,$x_{0}$itself is, indeed, the global minimum${\hat{R}}_{\text{KM}}$. Thus, we have

$$E_{f}=\frac{x_{0}}{R_{\text{KM}}^{*}t^{\text{*}}}=\frac{\beta^{-1}\left(R_{\text{KM}}^{*}t^{*}\right)}{R_{\text{KM}}^{*}t^{*}}=V\left(R_{\text{KM}}^{*}t^{*}\right),$$
(A. 31)

which confirmsEquation 9.
